# Biodegradable Polyester–Starch Composite Films Functionalized with Phenolic Compounds: Advances, Challenges, and Prospects for Sustainable Active Packaging

**DOI:** 10.3390/polym18121437

**Published:** 2026-06-09

**Authors:** Bongsoo Shin, Ajit Kumar Singh, Nattinee Bumbudsanpharoke, Seonghyuk Ko

**Affiliations:** 1 Laboratory of Nano-Enabled Packaging and Safety, Department of Packaging & Logistics, Yonsei University, Yonseidaegil, Wonju-si 26493, Republic of Korea; avodah@yongbong.net; 2 Research and Development Department, YONGBONG Co., Ltd., Incheon 22850, Republic of Korea; 3 Department of Packaging and Materials Technology, Faculty of Agro-Industry, Kasetsart University, Bangkok 10900, Thailand; nattinee.bu@ku.ac.th

**Keywords:** biodegradable polyesters, thermoplastic starch, phenolic compounds, active packaging, food safety, antioxidants, antimicrobials, biodegradability, sustainability

## Abstract

The growing demand for sustainable food packaging has intensified interest in biodegradable materials that can reduce environmental impact while preserving food quality. Among these materials, biodegradable polyester–starch composite films functionalized with phenolic compounds have gained attention as promising active packaging systems. They combine the melt processability and structural stability of polyesters, such as poly(lactic acid) (PLA), poly(butylene adipate-co-terephthalate) (PBAT), poly(butylene succinate) (PBS), and poly(3-hydroxybutyrate-co-3-hydroxyvalerate) (PHBV) with the renewability and biodegradability of starch and the antioxidant, antimicrobial, and UV-protective functions of phenolics, such as ferulic acid, quercetin, tea polyphenols, and anthocyanins. This review discusses recent advances in the selection of biodegradable polyesters, starch and thermoplastic starch blending, phenolic incorporation strategies, and their effects on compatibility, morphology, mechanical strength, barrier properties, optical behavior, release, and active packaging functionality. The characteristics and functionality of these films are governed not only by the individual components but also by phase morphology, interfacial interactions, phenolic location, processing conditions, and release control. Key challenges include polyester–starch incompatibility, TPS moisture sensitivity, phenolic stability during melt processing, migration safety, controlled release, and industrial scale-up. Collectively, biodegradable polyester–starch films functionalized with phenolic compounds represent a promising route for developing next-generation sustainable active packaging and may contribute to circular economy approaches.

## 1. Introduction

**Figure 1:**
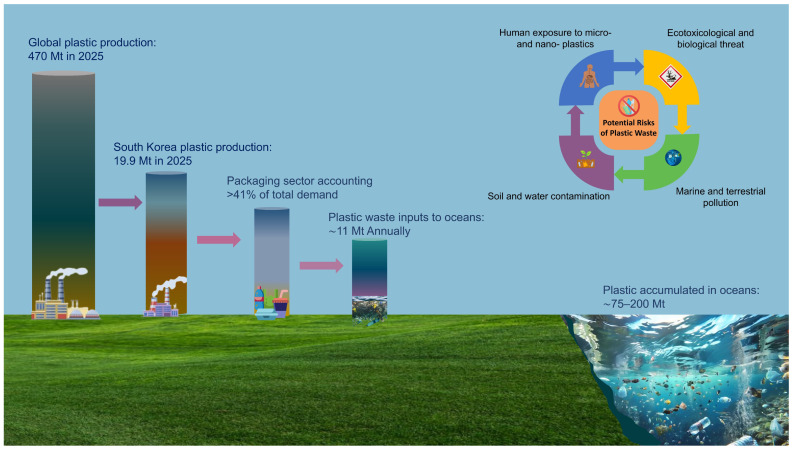
Overview of global and South Korean plastic production, packaging demand, and the major environmental risks associated with plastic waste accumulation.

Packaging is often the first point of contact through which consumers perceive the quality, safety, and acceptability of food products. It plays a vital role in protecting food from physical, chemical, microbiological, and environmental factors that can reduce quality and safety during handling and distribution. The global packaged food market was valued at US$1.9 trillion in 2020 and is expected to reach US$3.4 trillion by 2030, with an annual growth rate of 5% [1]. In parallel with the expansion of ready-to-eat, heat-and-eat, and grab-and-go foods, the packaging sector continues to rely heavily on plastic materials because of their light weight, durability, versatility, and relatively low cost. Among packaging materials, plastics account for approximately 37% of the global packaging market, followed by paper and cardboard (34%), glass (11%), and metals (6%) [2]. Plastic production has also increased in response to industrial growth and expanding demand, with food packaging remaining one of its largest application sectors. In 2025, global plastic production was estimated to reach 470 million metric tons, reflecting an average yearly growth of 4% from 2020 to 2025. According to Dokl et al. [3], approximately half of all plastics were produced in the past 15 years. Notably, packaging accounted for 146 million metric tons globally in 2025, the highest value among all end-use sectors. As illustrated in Figure 1, the continued increase in plastic production, together with the large share of packaging in the overall plastic demand, has intensified concerns regarding waste accumulation and its environmental consequences.

Although plastic packaging materials offer numerous benefits, their production, use, and disposal mostly follow a linear model. This creates major concerns for manufacturers, consumers, and governments because of their single-use nature and the challenges associated with their end-of-life management [4]. As shown in Figure 1, this linear plastic flow is closely associated with plastic leakage into natural environments. This is particularly evident in marine environments, where persistent plastic accumulation contributes to ecological disruption and is closely linked to broader concerns regarding soil and water contamination, ecotoxicological effects, and human exposure to micro- and nanoplastics. In addition, excessive production, irresponsible usage, and improper disposal of plastics have led to widespread plastic pollution and its harmful effects on human health and the environment [5,6]. Packaging waste also represents a considerable share of municipal solid waste, accounting for approximately 15–20% in many countries, and remains a serious challenge to environmental sustainability [7]. The Organisation for Economic Co-operation and Development (OECD) Global Plastics Outlook 2022 report indicates that packaging accounts for 37%, 38%, and 45% of total plastic waste in the United States, Europe, and China, respectively. Collectively, these regions contribute approximately 60% of the global packaging waste generation [8,9]. These concerns have increased interest in packaging materials with lower environmental impact, commonly referred to as sustainable packaging. The Sustainable Packaging Coalition (SPC) defines sustainable packaging as packaging that delivers value throughout its life cycle while minimizing environmental impacts through responsible sourcing, efficient material use, and effective recovery within circular systems [10,11,12].

In this context, biodegradable polyesters have emerged as promising candidates for sustainable packaging because they can potentially replace conventional plastics while offering biodegradability, film-forming ability, melt processability, and mechanical properties required for food packaging applications. These polyesters are characterized by hydrolyzable ester bonds in their backbone, making them susceptible to degradation under suitable environmental conditions via hydrolytic and microbial processes [13]. Polymers such as poly(lactic acid) (PLA), poly(butylene adipate-co-terephthalate) (PBAT), poly(butylene succinate) (PBS), and polyhydroxyalkanoates (PHAs) have attracted considerable attention as sustainable packaging materials. However, these polyesters have limitations, such as high cost, brittleness, lack of flexibility, and inadequate barrier properties, which may not be suitable for the specific packaging requirements of food products [14]. In this context, blending biodegradable polyesters with starch, cellulose, and other biopolymers is an important strategy for improving their key properties and sustainability [15,16]. Among these natural biopolymers, starch is particularly popular because of its abundance, low cost, renewability, and biodegradability. The inclusion of starch can thus decrease costs and enhance the renewable content of the packaging matrix, whereas the polyester phase aids in maintaining the melt processability, structural integrity, and moisture resistance of the packaging matrix [17]. Consequently, polyester–starch blends have been extensively investigated as an effective method for modifying the mechanical and barrier characteristics while preserving or improving the biodegradability of the final packaging material [18,19].

Beyond blending strategies aimed at improving barrier, mechanical, and thermal properties, recent packaging research has increasingly focused on incorporating active functionality into biodegradable polymer systems to extend shelf life and maintain food quality beyond the passive roles of conventional packaging materials. Active packaging is designed to interact with the packaged food or its surrounding environment to maintain or improve its condition. According to Commission Regulation (EC) No. 450/2009, active materials and articles function by deliberately releasing substances into or absorbing substances from packaged food or its surrounding environment [20]. Active packaging systems primarily function through two main mechanisms: absorbers or scavengers that remove undesired compounds, and releasers or emitters that release beneficial substances in a controlled manner [21]. Common examples include oxygen scavengers, carbon dioxide emitters or absorbers, ethylene scavengers, moisture absorbers, antioxidant packaging, and antimicrobial packaging materials. In this context, various active agents, including silver, copper nanoparticles, zinc oxide, and titanium dioxide, have been incorporated into packaging systems. However, concerns regarding migration, toxicological safety, regulatory acceptance, and consumer perception have intensified the interest in naturally derived alternatives [22]. Among these, phenolic compounds have emerged as promising multifunctional additives because they can impart antioxidant, antimicrobial, and UV-blocking properties while supporting sustainability goals in food packaging [21,23].

Despite the growing research interest in this area, current knowledge remains dispersed across several partially overlapping themes, including biodegradable polyesters, starch-based packaging materials, thermoplastic starch–polyester blends, and phenolic compounds used in active packaging. Previous reviews have addressed these topics separately; however, a focused and critical synthesis of biodegradable polyester/thermoplastic starch composite films functionalized with phenolic compounds is still lacking. This gap is significant because the key scientific questions are integrative rather than isolated, encompassing not only the selection of polymer matrices and phenolic additives but also their effects on blend compatibility, intermolecular interactions, morphology, mechanical and barrier properties, biodegradability, and packaging performance under realistic application conditions. In this context, the present review aims to advance the field by systematically examining these materials as a distinct class of sustainable active packaging systems. Particular emphasis is placed on the rationale for blending biodegradable polyesters with thermoplastic starch, the multifunctional role of phenolic compounds, their effects on key film properties, and the main challenges related to migration, processing stability, end-of-life behavior, and practical application in sustainable food packaging.

## 2. Biodegradable Polyesters for Sustainable Packaging

Biodegradable polyesters have emerged as one of the most important classes of polymer materials for sustainable food packaging because they combine degradability and thermoplastic processability. These characteristics are particularly advantageous for industrial conversion into films, sheets, coatings, trays, and molded products [24]. Compared with naturally derived polymers such as starch, cellulose, chitosan, and proteins, biodegradable polyesters generally offer a wider thermoplastic processing window and can be converted using conventional techniques such as extrusion, film blowing, thermoforming and injection molding [25]. Therefore, the combination of biodegradability, film-forming ability, and compatibility with traditional polymer processing techniques makes them attractive alternatives in the shift from enduring non-degradable plastics. However, biodegradable polyesters do not constitute a homogeneous class of materials. Their thermal behavior, crystallinity, stiffness, ductility, and barrier properties vary significantly, indicating that polyester selection is particularly important for food packaging applications [26].

### 2.1. Classification and Properties

The classification of biodegradable polyesters is significant because it provides a rational basis for selecting materials according to their source, structure, and degradation behavior. This facilitates the alignment of polyesters with their specific applications in packaging, agriculture, or biomedical fields, where the demands for durability and degradation vary significantly among different applications. As shown in Table 1, biodegradable polyesters can be broadly classified according to their origin into synthetic and microbial biodegradable polyesters [27]. Synthetic biodegradable polyesters are chemically prepared from renewable or petrochemical feedstocks. Common examples include polylactic acid (PLA), poly(glycolic acid) (PGA), poly(butylene succinate) (PBS), poly(butylene succinate-co-adipate) (PBSA), polycaprolactone (PCL), and poly(butylene adipate-co-terephthalate) (PBAT). In contrast, microbial biodegradable polyesters are produced by microorganisms and are mainly represented by the polyhydroxyalkanoate (PHA) family, including poly(3-hydroxybutyrate) (PHB), poly(3-hydroxyvalerate) (PHV), and poly(3-hydroxybutyrate-co-3-hydroxyvalerate) (PHBV) [28,29]. The second useful basis is their chemical structure, where they are grouped into aliphatic polyesters and aliphatic–aromatic copolyesters. Aliphatic polyesters, including PLA, PGA, PBS, PBSA, and PCL, have been widely studied because their ester linkages are generally more susceptible to hydrolysis. Aliphatic–aromatic copolyesters, such as PBAT, combine biodegradability with improved thermal and mechanical properties. In general, bio-based systems such as PLA and PHAs are attractive because of their renewable origin, whereas PBAT, PBS, PBSA, and PCL are valued primarily for their flexibility, melt processability, and suitability for blending [15,30].

Biodegradable polyesters exhibit a wide range of tunable thermal, mechanical, barrier, and degradation properties, making them suitable for diverse applications in various fields. Key characteristics include the glass transition temperature (*T*_g_), melting temperature (*T*_m_), mechanical properties such as tensile strength and elongation at break (EAB), barrier properties, and susceptibility to hydrolytic and enzymatic degradation via ester linkages [31]. The suitability of biodegradable polyesters for sustainable food packaging is governed by a combination of these properties, as summarized in Table 2. These materials do not exhibit uniform properties. Each polyester presents a distinct balance of stiffness, ductility, thermal resistance, and gas/moisture permeability, which determines its suitability for rigid containers, semi-rigid trays, flexible films, and high-barrier multilayer packaging systems. Therefore, the selection of a suitable biodegradable polyester depends on the specific requirements of the packaged food and the desired packaging format, in addition to biodegradability [32].

Among the commonly used biodegradable polyesters, PLA is one of the most extensively studied because of its relatively high tensile strength (20–75 MPa), *T*_g_ (55–65 °C), and *T*_m_ (148–166 °C) [33,34]. These properties endow PLA with excellent stiffness, dimensional stability, and optical clarity. However, its low EAB (2–12%) reflects its intrinsic brittleness, limiting its use in highly flexible applications. PLA also exhibits moderate water vapor permeability (1–5 g·mm/m^2^·day) and oxygen permeability (18–25 cm^3^·mm/m^2^·day) [35]. This makes it suitable for a wide range of packaging applications, including food packaging. However, these properties may not be sufficient for foods that are highly sensitive to moisture and oxygen [33]. Furthermore, PGA exhibits the highest density (1.53 g/cm^3^) and *T*_m_ (220–230 °C) among biodegradable polyesters, as shown in Table 2. In addition, it demonstrates very high tensile strength (69–115 MPa) and exceptionally low oxygen permeability (0.2–0.3 mL/m^2^·day·bar) [36,37]. These characteristics make PGA particularly attractive for high-barrier-packaging applications. Nevertheless, its low EAB (3–4%), high crystallinity, and narrow processing window render it relatively brittle and more difficult to process than other flexible biodegradable polyesters [38,39,40].

PBS and PBSA represent a more flexible class of aliphatic biodegradable polyesters. PBS exhibits a relatively low *T*_g_ (−36 to −33 °C), moderate *T*_m_ (104–113 °C), tensile strength of 30–50 MPa, and an EAB of 15–185% [35,41,42]. This combination provides a favorable balance of strength, flexibility, and processability, which explains its relevance to film, sheet, and flexible packaging applications [43,44]. PBSA exhibits even higher ductility, with EAB values exceeding 300% and a lower *T*_m_ (86–97 °C), making it highly attractive for flexible film processing [41,45]. However, PBSA generally exhibits poor barrier properties compared to more crystalline high-barrier biodegradable polyesters, thereby restricting its use in applications that require strict control of moisture or oxygen transfer [41,42,45].

**Table 1:** Classification of major biodegradable polyesters based on their group, subgroup, structure, molecular formula, and typical origin.

Main Group	Subgroup	Polymer	Full Name	Structure	Molecular Formula	Typical Origin
Synthetic biodegradable polyesters	α-Hydroxy-acid polyesters	PLA	Poly(lactic acid)	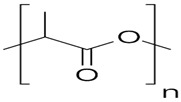	(C_3_H_4_O_2_)_n_	Mainly bio-based
		PGA	Poly(glycolic acid)	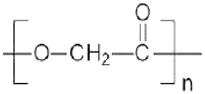	(C_2_H_2_O_2_)_n_	Bio-based or fossil-based
Synthetic biodegradable polyesters	Succinate-based aliphatic polyesters	PBS	Poly(butylene succinate)	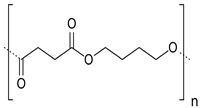	(C_8_H_12_O_4_)_n_	Bio-based or fossil-based
		PBSA	Poly(butylene succinate-co-adipate)	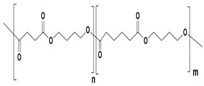	(C_8_H_12_O_4_)_m_ (C_10_H_16_O_4_)_n_	Bio-based or fossil-based
Synthetic biodegradable polyesters	Lactone-based aliphatic polyesters	PCL	Poly(ε-caprolactone)	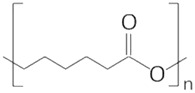	(C_6_H_10_O_2_)_n_	Mainly fossil-based
Synthetic biodegradable polyesters	Aliphatic–aromatic biodegradable copolyesters	PBAT	Poly(butylene adipate-co-terephthalate)	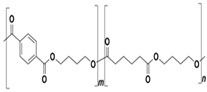	(C_10_H_16_O_4_)_m_ (C_12_H_12_O_4_)_n_	Mainly fossil-based
Microbial biodegradable polyesters	Polyhydroxyalkanoates (PHAs)	PHB	Poly(3-hydroxybutyrate)	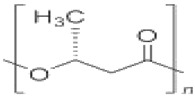	(C_4_H_6_O_2_)_n_	Bio-based
		PHV	Poly(3-hydroxyvalerate)	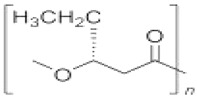	(C_5_H_8_O_2_)_n_	Bio-based
		PHBV	Poly(3-hydroxybutyrate-co-3-hydroxyvalerate)	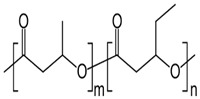	(C_4_H_6_O_2_)_m_ (C_5_H_8_O_2_)_n_	Bio-based

**Table 2:** Common biodegradable polyesters, key properties, and characteristics for sustainable packaging applications.

Polymer	Density (g/cm^3^)	*T*_g_ (°C)	*T*_m_ (°C)	Tensile Strength (MPa)	EAB (%)	WVP/WVTR	OP/OTR	Characteristics	References
PLA	1.23–1.25	55–65	148–166	20–75	2–12	1–5 ^a^	18–25 ^b^	High mechanical strength and stiffness; good optical clarity; brittle; moderate barrier properties; biodegradable; mainly industrially compostable	[33,34,35]
PGA	1.53	35–40	220–230	69–115	3–4	2.9–3.4 ^c,d^	0.2–0.3 ^e,f^	Excellent gas barrier and high mechanical strength; high crystallinity; brittle; narrow processing window; biodegradable; compostable	[36,37,38,39,40,46]
PBS	1.23–1.26	−36 to −33	104–113	30–50	15–185	5–15 ^a^	4–30 ^b^	Good balance of strength and flexibility; good processability; biodegradable; compostable; suitable for films, sheets, and flexible packaging	[35,41,42,43,44]
PBSA	1.23–1.24	−45 to −40.9	86–97	21–22	300–314	~800 ^c^	3.8 × 10^4 g^	High ductility and good processability; low melting temperature; relatively low barrier properties; biodegradable; compostable; suitable for flexible packaging	[41,42,45,47]
PCL	1.07–1.20	−65 to −50	55–60	24–33	200–450	565–783 ^c,h^	485.7–1457.3 ^i,j^	High flexibility and toughness; low glass-transition and melting temperatures; easy processing; poor gas and water-vapor barrier properties; biodegradable; suitable for flexible and blend-based packaging	[35,48,49,50]
PBAT	1.25–1.27	−28 to −23	110–120	12–21	350–700	173–177 ^c^	1179–1181 ^k^	High flexibility and toughness; low glass-transition temperature; good processability; relatively low oxygen and water-vapor barrier compared with high-barrier packaging polymers; biodegradable; suitable for flexible packaging and blending applications	[51,52,53,54]
PHB	1.18–1.26	4–10	170–180	15–40	4–10	0.30–0.98 ^a^	2–10 ^l^	High crystallinity and stiffness; good oxygen barrier; brittle; narrow processing window; biodegradable; compostable; suitable for rigid articles, coatings, and high-barrier packaging applications	[55,56,57]
PHBV	1.24–1.25	−8 to −3	165–176	22–39	2–5	12–15 ^c^	20–21 ^i^	High crystallinity and good oxygen/moisture barrier; rigid and brittle; narrow processing window; biodegradable; compostable; suitable for high-barrier and coating-based packaging applications	[58,59,60,61]

*T*_g_, glass transition temperature; *T*_m_, melting temperature; EAB, elongation at break; WVP, water vapor permeability; WVTR, water vapor transmission rate; OP, oxygen permeability; OTR, oxygen transmission rate; ^a^ WVP, g·mm/m^2^·day; ^b^ OP, cm^3^·mm/m^2^·day; ^c^ WVTR, g/m^2^·day; ^d^ measured at 23 °C/85% RH; ^e^ OTR, mL/m^2^·day·bar; ^f^ measured at 25 °C/90% RH; ^g^ cm^3^·µm/(m^2^·day); ^h^ measured at 23 °C/65% RH for neat PCL films of 40–60 μm thickness; ^i^ OTR, cm^3^/m^2^·day; ^j^ measured at 5 °C and 23 °C under dry conditions for neat PCL films of 40–60 μm; ^k^ OTR, cm^3^/m^2^·day·atm; ^l^ OP, cm^3^·mm/m^2^·day·atm.

PCL and PBAT are particularly valued for their flexibility, toughness, and ease of processing. PCL possesses a very low *T*_g_ (−65 to −50 °C) and low *T*_m_ (55–60 °C), which contribute to its high extensibility (200–450%) and excellent melt processability [35,50]. However, PCL generally exhibits poor gas and water vapor barrier properties, limiting its standalone use in demanding food packaging applications [48,49,50]. Similarly, PBAT has excellent flexibility, characterized by a low *T*_g_ (−28 to −23 °C), moderate *T*_m_ (110–120 °C), relatively low tensile strength (12–21 MPa), and an exceptionally high EAB (350–700%) [51,52,53]. These characteristics position PBAT as one of the most important biodegradable polyesters for flexible packaging and compatibilized blends. Despite the remarkable flexibility and processability of PBAT, its main limitation is its moderate barrier properties compared to those of high-barrier packaging polymers [51,62].

Within the microbial polyester family, PHB and PHBV are distinguished by their high crystallinity and strong barrier properties. PHB exhibits low water vapor permeability and favorable oxygen barrier properties, along with tensile strength (15–40 MPa) and *T*_m_ (170–180 °C) [55,56,58]. PHBV exhibits similar thermal behavior and provides good oxygen and moisture barrier properties [59]. However, both PHB and PHBV generally suffer from low EAB (2–10%) and inherent brittleness, which restricts their direct use in applications requiring toughness and flexibility. Despite their clear advantages for rigid, coating-based, and high-barrier packaging applications, their narrow processing windows complicate large-scale conversion [56,61].

### 2.2. Biodegradable Polyester Blends

**Figure 2:**
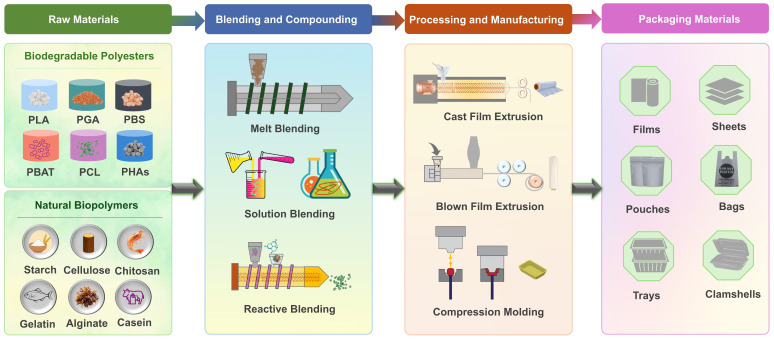
Schematic illustration of biodegradable polyester blend systems from raw materials to sustainable packaging materials.

Biodegradable polyester blends have become a promising approach for developing sustainable packaging materials because they make it possible to combine properties that are difficult to obtain from a single polymer alone. By combining two or more biodegradable polyesters or a biodegradable polyester with another biopolymer, blending provides a versatile route to overcome the intrinsic limitations of individual materials, such as brittleness, limited thermal resistance, poor barrier properties, and high cost [15,34]. In many cases, the optimal balance of strength, flexibility, thermal stability, processability, barrier performance, and biodegradability required for practical packaging applications is hard to achieve using a single biodegradable polyester. Therefore, blending has become an important strategy for designing biodegradable materials with improved structure and key properties. The performance of these blends depends on several factors, including miscibility, interfacial adhesion, phase morphology, rheological compatibility, and crystallization behavior, which influence the final mechanical, thermal, and barrier properties [63]. Depending on the chemistry of the constituent polymers, biodegradable polyester blends may be immiscible, partially miscible, or improved via physical or reactive compatibilization. Their properties can also be tailored using different processing methods, such as melt blending, solution blending, and reactive blending. Owing to this flexibility, blending has become an important approach in the development of biodegradable packaging materials for applications ranging from flexible films to rigid trays and barrier laminates [64,65]. Figure 2 schematically illustrates the biodegradable polyester blending route from raw materials to sustainable packaging materials, highlighting the major blend constituents, blending approaches, processing techniques, and representative packaging products.

In this context, several biodegradable polyester blend systems have been investigated to improve their toughness, processability, barrier properties, and biodegradation behavior. For instance, Yang et al. [66] developed a three-layer co-extruded PLA/PBAT composite film for packaging applications, in which the rigidity of PLA was combined with the flexibility of PBAT, resulting in an improved oxygen barrier and optimal mechanical properties. Similarly, Zhang et al. [67] reported on PLA/PCL blended films for strawberry packaging, in which the addition of PCL improved the compatibility, flexibility, and water vapor barrier properties owing to the smaller dispersed-phase size and stronger interfacial interactions within the blend. Shin et al. [68] developed PBAT/PGA blended films for sustainable packaging and demonstrated that the addition of PGA improved the mechanical strength, barrier properties, and degradation behavior of the blend. These examples demonstrate that blending biodegradable polyesters is an effective method for developing materials with improved properties for specific applications. However, polyester–polyester blend systems still have some important limitations, such as incomplete miscibility, weak interfacial adhesion, phase separation, property instability during processing and storage, and the relatively high cost of most biodegradable polyesters. These challenges continue to limit their commercial use and highlight the need for more economical and sustainable blending strategies. In practical packaging films, such structural defects can reduce stress transfer, promote crack initiation, create unstable diffusion pathways for gases and moisture, and lead to property changes during processing or storage [30,69].

In this regard, blending biodegradable polyesters with natural biopolymers is an attractive alternative for developing sustainable packaging materials. Natural biopolymers such as starch, cellulose, chitosan, and proteins offer several advantages because they are renewable, abundant, inexpensive, environmentally friendly, and biodegradable [70]. Among them, starch has received significant attention as a blending component because it is broadly available, inexpensive, and inherently biodegradable. When plasticized as thermoplastic starch, it can also be processed more effectively in polymer blend systems. This makes it particularly useful for lowering formulation costs and increasing the renewable content of polyester-based materials. At the same time, biodegradable polyesters can help overcome the poor processability, low moisture resistance, and limited mechanical strength of starch-based systems. Consequently, starch blending has become an efficient, practical, and cost-effective strategy for developing biodegradable materials with tailored thermal, mechanical, barrier, and biodegradation properties, although the final performance depends strongly on compatibility and formulation design [71,72].

## 3. Biodegradable Polyester–Starch Blends

Recently, biodegradable polyester–starch blends have gained significant interest as promising sustainable packaging materials. These blends combine the processability of biodegradable polyesters with the renewability, biodegradability, and low cost of starch. Polyesters such as PLA, PBAT, PCL, and PHAs provide mechanical strength, barrier properties, and easier processing, whereas starch helps increase the renewable content and reduce material costs. This makes such blends particularly attractive for short-life and disposable-packaging applications. However, these systems are not simple because starch is hydrophilic, whereas many biodegradable polyesters are relatively hydrophobic, which often leads to poor interfacial compatibility, phase separation, and unstable properties if the formulation is not properly controlled. Their blending behavior is strongly influenced by the form of starch used, whether native, modified, or thermoplastic starch. It also depends on the degree of phase dispersion and interfacial adhesion achieved during the processing. Therefore, understanding the role of starch is important before discussing thermoplastic starch formation, blend morphology, and strategies for improving its performance.

### 3.1. Starch as a Renewable Blending Component

Starch is a promising component of sustainable polymeric materials and is widely recognized as a readily available natural resource for developing packaging materials with renewable, biodegradable, safe, and economical characteristics. Many conventional sources, such as corn, wheat, rice, and potatoes, as well as non-conventional and underutilized sources, including beans, peas, lentils, chestnuts, amaranth (*Amaranthus hypochondriacus*), sago palm (*Metroxylon sagu*), and quinoa (*Chenopodium quinoa*), are used as diverse sustainable sources of starch for packaging materials [6]. These features make starch particularly appealing as a blending component for biodegradable polyesters, as it helps reduce the formulation cost while increasing the bio-based content. Starch is also suitable for packaging applications, specifically in films and coatings, because it can provide good transparency and favorable gas barrier properties, particularly against oxygen and carbon dioxide, under suitable conditions [19,73]. Thus, its renewability, biodegradability, wide availability, and low cost provide starch with a clear advantage over many other natural biopolymers for sustainable packaging applications.

Furthermore, the significance of starch in polyester-based blends extends beyond cost reduction. Because starch contains many hydroxyl groups and has a strong affinity for water, its incorporation can increase the polarity, water sensitivity, biodegradation tendency, and morphological complexity of polyester blends [71]. While the addition of starch can improve renewable content and facilitate degradation, it may also reduce moisture resistance and weaken interfacial adhesion with hydrophobic polyester phases [18,71]. Thus, starch should be regarded not only as a low-cost filler but also as a structural component that strongly influences the mechanical and barrier properties of polyester–starch films. Consequently, the design of these blends requires careful control of composition, plasticization, dispersion, and compatibilization [15,18].

Despite these challenges, starch remains particularly valuable in sustainable packaging because it allows the development of materials that are more economically and environmentally attractive than neat biodegradable polyesters. Many recent studies on polyester–starch blend systems have shown that starch can help produce packaging films and related materials with modified stiffness, flexibility, biodegradation behavior, and, when properly compatibilized, acceptable processability and barrier properties of the packaging films [17]. For example, Wang et al. [74] prepared starch-based biodegradable composite films composed of thermoplastic starch, PLA, and PBAT using extrusion followed by in situ re-extrusion and showed that repeated extrusion refined the TPS-rich phase, altered crystallinity, and influenced the mechanical and surface properties of the films. Notably, two re-extrusion cycles improved elongation at break, Young’s modulus, and water contact angle, although excessive re-extrusion reduced thermal stability and weakened tensile performance. In another study, Wongphan et al. [72] developed PBAT/thermoplastic cassava starch blown films and demonstrated that after starch was plasticized into thermoplastic starch (TPS), it could be effectively processed by melt extrusion and film blowing. The resulting films exhibited better compatibility, a smoother morphology, and improved oxygen barrier properties. These studies demonstrate the importance of starch as a widely studied natural blending component in biodegradable polyester systems and emphasize the key role of TPS in sustainable packaging applications.

### 3.2. Thermoplastic Starch in Biodegradable Polyester Blends

**Figure 3:**
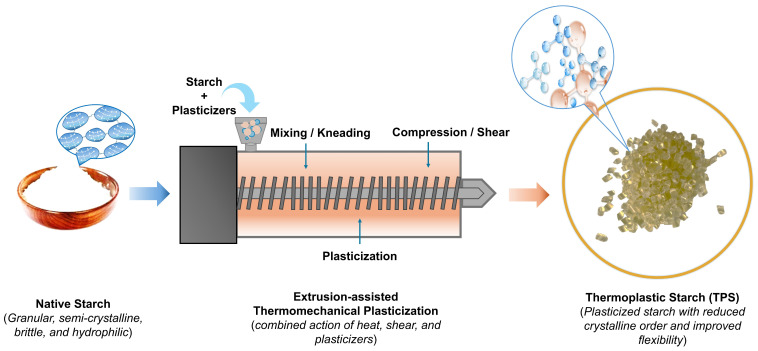
Extrusion processing of native starch toward thermoplastic starch.

Native starch is a semi-crystalline, hydrophilic, and brittle material that is not directly suitable for most thermoplastic processing operations because of its granular structure, extensive intermolecular hydrogen bonding, and tendency to degrade before melt processing. Accordingly, starch is commonly transformed into thermoplastic starch (TPS) through the combined action of heat (90–180 °C), shear, and plasticizers, such as water, glycerol, and sorbitol (Figure 3) [75]. Thermomechanical plasticization disrupts the native semi-crystalline granule structure, reduces the intermolecular association among starch chains, and converts starch into a processable thermoplastic phase that can be blended with biodegradable polyesters via extrusion and related melt-processing methods. Consequently, TPS has become the most widely used starch form in the development of biodegradable packaging materials because it enables starch incorporation into polymer-processing routes that are suitable for film and sheet fabrication [76].

The importance of TPS in biodegradable polyester blends lies in its ability to connect the low cost and renewability of starch with the melt processability and structural stability of biodegradable polyesters. In packaging systems based on PLA, PBAT, PBS, PBSA, and related copolyesters, TPS acts as a renewable dispersed or continuous phase, whereas the polyester matrix provides flexibility, cohesion, and improved moisture resistance. For example, Dhamvithee et al. [77] employed TPS as a renewable and melt-processable phase in PLA-based composites, and its plasticized structure allows blending through melt extrusion and increases ductility and water uptake compared to neat PLA. Their study also showed that TPS could be incorporated more effectively into a polyester matrix when PLA-grafted glycidyl methacrylate (PLA-g-GMA) was used as a compatibilizer. This compatibilizer improved interfacial bonding through epoxy ring-opening reactions with the hydroxyl groups of TPS, which enhanced phase dispersion and blend integrity. Similarly, Morinval et al. [78] demonstrated that TPS and polyester blends can be processed into multilayer systems by coextrusion, with thermoplastic starch contributing to the oxygen barrier properties and polyester outer layers improving the thermal stability and water vapor resistance. These studies demonstrate that TPS is not simply an economic biopolymer component but a significant processable functional phase in designing biodegradable polyester blends.

Despite these advantages, polyester blends containing TPS still present critical technical challenges. Their performance is strongly affected by the type and amount of plasticizer, residual moisture, retrogradation, and polarity difference between the hydrophilic thermoplastic starch and hydrophobic polyester phases [18]. Without compatibilization, formulation control, or multilayer design, these systems often exhibit weak interfacial adhesion, phase separation, moisture sensitivity, and unstable mechanical or barrier properties. Therefore, the effective use of TPS in biodegradable polyester blends depends not only on starch plasticization but also on the proper control of the morphology and interfacial structure during processing [18,19].

### 3.3. Compatibility and Morphology of Biodegradable Polyester–Starch Blends

Compatibility and morphology are among the most important factors that determine the characteristics of biodegradable polyester–starch blends. In most cases, these systems are intrinsically incompatible because starch or TPS is hydrophilic and contains many hydroxyl groups, whereas many biodegradable polyesters are relatively hydrophobic. This difference in polarity often leads to weak interfacial adhesion, phase separation, and non-uniform morphology [69]. As a result, mechanical strength and barrier properties are often reduced. Therefore, the effective blending of the polyester–starch system depends strongly on the degree of interfacial interaction, phase dispersion, and morphological stability achieved during processing [18]. Morphologically, biodegradable polyester–starch blends exhibit different phase structures, including starch domains dispersed in a continuous polyester matrix, phase inversion, or co-continuous morphologies. These structures depend on the blend composition, degree of starch plasticization, viscosity ratio, and processing conditions [79]. Poorly compatibilized systems typically exhibit coarse phase separation, starch agglomeration, interfacial voids, and rough fractured surfaces. These features indicate weak interfacial interactions and inefficient stress transfer between the phases. In contrast, improved compatibility is usually reflected by a reduced domain size, smoother microstructure, better dispersion of the starch-rich phase, and more coherent interfacial contact between the two components [80].

In addition to chemical compatibilization, physical strategies also play a vital role in controlling polyester–starch blend morphology. Processing parameters, such as melt temperature, shear intensity, screw speed, residence time, moisture level, and viscosity ratio, can affect TPS dispersion, starch agglomeration, and the formation of dispersed or co-continuous structures [78]. Multilayer coextrusion provides another physical approach, where starch/TPS-rich layers can contribute to oxygen barrier properties and renewable content, while polyester-rich outer layers can improve moisture resistance, thermal stability, and handling properties. However, this strategy requires careful control of interlayer adhesion, rheological matching, layer stability, and processing complexity [79,81].

In this context, the compatibility of biodegradable polyester–starch blends can be improved by reducing the polarity mismatch between phases, lowering the interfacial tension, and strengthening the interphase through physical or chemical interactions [79,81]. In principle, effective compatibilization promotes finer phase dispersion, suppresses starch agglomeration and interfacial voids, and improves stress transfer across the matrix–dispersed phase boundary. Non-reactive compatibilization approaches often rely on hydrogen-bond-driven interactions or starch modification. For example, Wadaugsorn et al. [82] showed that the hydroxypropylation of cassava starch improved its compatibility with PBAT by disrupting starch crystallinity, increasing chain mobility, and facilitating more homogeneous phase structures. Furthermore, Qin et al. [83] reported that pyrogallic acid acted as a plant-derived compatibilizer in PLA/TPS blends, where multiple hydroxyl groups promoted intermolecular interactions, leading to improved morphology, tensile strength, crystallinity, thermal stability, and hydrophobicity of the blends. Similarly, Bulatović et al. [84] found that low levels of citric acid improved the morphology of TPS/PLA blends by promoting esterification with starch hydroxyl groups and modifying the interfacial tension, although excessive citric acid caused agglomeration, void formation, and poorer miscibility.

Reactive compatibilization is often more effective because functional additives can form covalent bonds at the interface between the polyester and starch phases [69]. Common reactive compatibilizers include epoxy-, anhydride-, and isocyanate-based additives, which can react with the hydroxyl groups of TPS and, in some systems, with the terminal groups of polyesters [18,85]. For instance, Dhamvithee et al. [77] demonstrated that PLA-g-GMA improved the dispersion and structural integrity of PLA/TPS composites through epoxy ring-opening reactions with TPS hydroxyl groups. In another study, Taghinezhad et al. [86] reported that poly(ethylene-co-glycidyl methacrylate) (EGMA) and maleic anhydride-grafted compatibilizers significantly reduced the dispersed domain size and greatly improved the ductility of TPS/PLA blends. In PBS/TPS systems, Patil et al. [87] further showed that citric acid and citric acid-modified castor oil polyurethane improved the morphology and flexibility during reactive extrusion. These studies indicate that interfacial reactions can be used to toughen polyester–starch films. Therefore, the compatibilization of polyester–starch systems does not rely on a single mechanism. Instead, it involves different approaches, including starch modification, hydrogen bonding, and reactive interfacial chemistry, to tailor the morphology and improve the mechanical, thermal, barrier, and biodegradation properties of the blended material [18,88].

Mechanistically, non-reactive compatibilization improves the interaction between polyesters and starch mainly by promoting hydrogen bonding, adjusting polarity, improving plasticization, modifying starch behavior, and lowering interfacial tension. In contrast, reactive compatibilization can create stronger interfacial bonding. This occurs mainly because of functional groups, such as epoxy, anhydride, carboxyl, or isocyanate groups, that can react with the hydroxyl groups of starch [18,69]. Furthermore, during blending, the continuous polyester phase usually provides better moisture resistance, mechanical strength, and film-forming stability, whereas TPS-rich or co-continuous systems can increase renewable content, improve oxygen barrier properties under dry conditions, support biodegradation, and allow better phenolic release. However, these systems are often more sensitive to water and dimensional changes [18,79]. Similarly, monolayer melt-compounded films are easier to process and more suitable for industrial scale-up, whereas multilayer or carrier-assisted systems can provide better functional separation and more controlled release. These advanced structures may also face challenges such as poor interlayer adhesion, higher processing complexity, increased cost, and reduced long-term storage stability [64,78].

## 4. Phenolic Compounds in Biodegradable Polyester–Starch Systems

In recent years, phenolic compounds have been increasingly explored as multifunctional additives in biodegradable polyester–starch systems. These materials can provide antioxidant and antimicrobial activities and, in some cases, can also act as crosslinking or compatibilization agents [21,89]. This makes them attractive for the development of sustainable active packaging materials. In contrast to many inorganic or nano-enabled active agents, phenolic compounds are naturally occurring, widely distributed in plant biomass, and increasingly available from food-processing residues and agricultural byproducts [90]. Their use in biodegradable polyester–starch films is particularly relevant because these systems require additives that not only provide active functionality but also interact favorably with hydrophilic starch-rich domains and biodegradable-polyester matrices. In such blends, low-molecular-weight phenolics, tannins, and phenolic-rich extracts can interact with both phases, mainly through hydrogen bonding and, in specific systems, through covalent interactions. Consequently, they can positively influence the morphology, mechanical strength, and barrier properties [91].

Phenolic compounds exhibit antioxidant activity primarily through hydrogen atom transfer, single-electron transfer, and metal-chelating mechanisms, collectively retarding oxidative degradation in food systems [92]. For example, Hernández-García et al. [93] developed active starch–polyester bilayer films containing ferulic acid and demonstrated that the incorporation of this phenolic acid provided antioxidant activity and helped preserve pork meat. This study effectively demonstrates the functional role of phenolic compounds in biodegradable polyester–starch-based active packaging systems. In addition to their antioxidant properties, phenolic compounds exhibit antimicrobial properties. This activity is generally related to their ability to interact with microbial cell membranes, increase membrane permeability, cause intracellular component leakage, and interfere with essential enzymes and metabolic functions. Therefore, they are particularly beneficial for active packaging films designed to control spoilage and pathogenic microorganisms. For example, Ordoñez et al. [94] developed multilayer antimicrobial films based on starch and PLA with superficially incorporated ferulic acid or cinnamic acid. They showed that these phenolic compounds exhibited antimicrobial activity, particularly against *Listeria innocua* and *Escherichia coli*. Their results also indicated that the antimicrobial effect depended on the method of incorporation, with electrospun active layers showing higher effectiveness because the phenolic acids were released more efficiently from the film surface. Table 3 summarizes recent studies on biodegradable polyester–starch films functionalized with phenolic compounds for sustainable active packaging applications.

### 4.1. Classification and Sources of Phenolic Compounds

**Figure 4:**
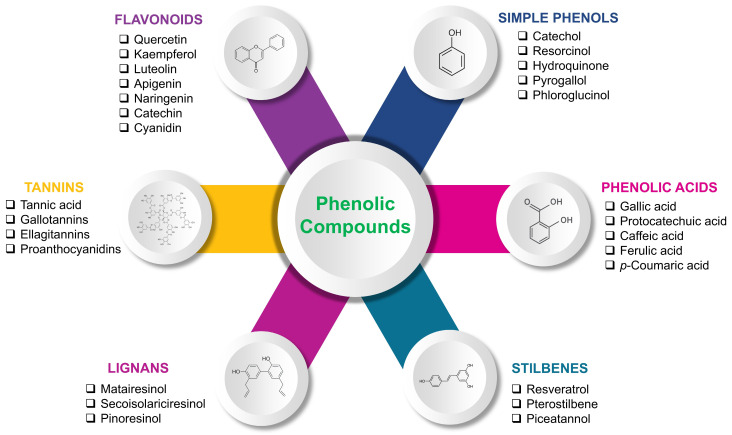
Overview of the major classes of phenolic compounds.

Phenolic compounds are a structurally diverse group of plant secondary metabolites characterized by the presence of one or more hydroxyl groups attached to aromatic ring structures. Their main natural sources include fruits, vegetables, herbs, grains, seeds, oils, tea, coffee, chocolate, wine, and numerous plant extracts, and they are commonly categorized according to the number of phenolic rings and the structural elements that link them [21,104]. As shown in Figure 4, the six main structural groups of phenolic compounds are flavonoids, phenolic acids, simple phenols, stilbenes, lignans, and tannins. Moreover, more than 10,000 phenolic compounds with diverse biological and functional properties have been identified, of which flavonoids account for approximately 60% and phenolic acids for approximately 30% [105]. In food packaging, phenolic compounds are valued mainly for their antioxidant and antimicrobial functions because they can extend shelf life by retarding lipid and pigment oxidation and limiting microbial growth on food surfaces or within the package headspace. They are also particularly attractive for active films and coatings because they can be incorporated into biodegradable matrices without relying on synthetic additives [21,106]. In polyester–starch active films, the suitable loading of phenolic compounds is formulation-dependent and varies with the phenolic structure, matrix polarity, incorporation method, and the required antioxidant or antimicrobial effect. Low-molecular-weight phenolic acids and flavonoids may be suitable for direct incorporation at controlled levels, whereas thermally sensitive compounds, particularly anthocyanins and some phenolic-rich extracts, are less suitable for direct high-temperature melt processing and may require surface loading, encapsulation, or multilayer delivery to preserve their functionality [93,96,98,103].

### 4.2. Incorporation of Phenolic Compounds into Biodegradable Polyester–Starch Films

The method used to incorporate phenolic compounds is an important design factor in biodegradable polyester–starch films because it affects phenolic retention during processing, spatial distribution within the matrix, release behavior, and the balance between the film properties and active function of the film. In practice, recent studies have mainly used four approaches: direct incorporation into the polymer blend during melt compounding or extrusion, incorporation into bilayer or multilayer structures by surface deposition, carrier-assisted loading through electrospun layers, and protected delivery through encapsulated or microencapsulated phenolic systems [93,95,97,103]. Thus, the selection of the method mainly depends on the physicochemical properties of the phenolic compound or extract, particularly its thermal stability, polarity, and release behavior, as well as the targeted packaging format and processing method [96,100].

Among these strategies, direct melt incorporation is particularly promising for industrial packaging because it is compatible with extrusion-blowing, sheet-extrusion, and other thermoplastic processing methods. In PBAT/starch systems, Zhai et al. [96] incorporated tea polyphenols into extrusion-blown films using a one-pot masterbatch approach, demonstrating that this method allowed for high phenolic retention while maintaining the feasibility of large-scale processing. In another study, the same group further showed that when tea polyphenols were incorporated into PBAT/TPS films, the starch-rich phase interacted strongly with the phenolic compounds through hydrogen bonding and acted as a hydrophilic regulator of swelling and release [97]. This finding suggests that these polymer structures can also be used to control functional release. Similarly, Yang et al. [98] incorporated quercetin into PBAT/TPS films via extrusion blowing and demonstrated that direct compounding is feasible even for low-molecular-weight phenolic compounds when the formulation and processing conditions are properly controlled. In PLA/starch systems, Li et al. [100] used pomegranate peel as a phenolic-rich additive in composite extrusion sheets and found that starch not only reduced the formulation cost but also promoted the release of bioactive compounds from the PLA matrix, thereby acting as a release-controlling phase.

Surface incorporation has also emerged as a critical approach, particularly for phenolic acids, which are effective at low concentrations but may be sensitive to prolonged thermal exposure or need to be rapidly available at the food-contact surface. For example, Hernández-García et al. [95] developed active starch–polyester bilayer films by spraying ferulic acid onto a polyester food-contact layer after bilayer formation. This approach avoided thermal degradation during bulk processing and preserved the barrier and mechanical properties of the film while providing antioxidant and antibacterial activity. A similar strategy was reported by Ordoñez et al. [94], who prepared PLA/starch/PLA trilayer films and compared two superficial incorporation methods for ferulic and cinnamic acids: solution spraying and electrospinning of phenolic-loaded PLA layers. Both methods produced active films; however, the electrospun layers were more effective because they allowed better phenolic encapsulation and release from the film surface. These studies show that multilayer design and superficial loading are useful when the objective is to preserve the structural role of the polyester–starch substrate while localizing phenolic functionality at the food-contact interface, where antioxidant or antimicrobial activity is most relevant for food preservation. Therefore, the selection of a phenolic incorporation strategy should not be based solely on active functionality, but also on the balance among thermal retention during processing, spatial distribution within the film, release kinetics, migration safety, and compatibility with industrial film manufacturing [93,97].

In recent years, biodegradable polyester–starch systems have been increasingly used in protected delivery approaches, especially for anthocyanins and other phenolic compounds that are sensitive to heat, oxidation, or uncontrolled migration. Although this method is still less common than direct compounding or surface deposition, microcapsule-based incorporation has become a useful strategy for improving phenolic stability during processing and achieving more controlled functional behavior in active and intelligent packaging films. This trend can be observed in recent PBAT/TPS systems containing anthocyanin microcapsules, where the films exhibit clear color changes in response to shrimp spoilage, allowing real-time freshness monitoring [103]. Therefore, the incorporation of phenolic compounds into biodegradable polyester–starch films should not be regarded as a simple additive step but rather as a formulation strategy in which the processing route, matrix polarity, and release requirements need to be co-designed to obtain stable and functional active packaging materials.

### 4.3. Properties and Active Packaging Functions of Phenolic-Functionalized Films

The incorporation of phenolic compounds into biodegradable polyester–starch films influences not only their active properties but also the underlying key physical properties of the packaging materials. As shown in Table 3, these systems reflect multifunctional matrices in which the film structure, phase morphology, interfacial interactions, and release behavior are influenced by the type of phenolic compound, its concentration, and the incorporation method [93,103]. In general, phenolic compounds can improve the oxygen and water vapor barrier properties, UV shielding, and antioxidant and antimicrobial activities. However, they may also reduce stiffness or extensibility when phase heterogeneity, crystallization changes, or excessive loading disrupts the polymer network. Therefore, the properties of phenolic functionalized polyester–starch films depend on the combined effects of composition, morphology, and interactions between the different components, rather than the presence of the bioactive additive alone [97,98].

From a materials perspective, phenolic compounds can strongly influence the mechanical, barrier, optical, and surface properties of biodegradable polyester–starch films. Hernández-García et al. [93] reported that PLA:PHBV/cassava starch–gellan bilayer films containing ferulic acid, p-coumaric acid, or protocatechuic acid showed notable improvements in barrier properties, with WVP reduced by approximately 53–65% and OP reduced by approximately 18–33%. During pork meat storage, these films reduced TBARS by approximately 21–33% after 15 days, while microbial counts decreased by up to 1.1 log CFU/g for total coliforms and 1.2 log CFU/g for lactic acid bacteria. However, the inclusion of these phenolic acids affected the mechanical properties, as TS decreased by approximately 25–33%. This result suggests a potential trade-off in phenolic-functionalized polyester–starch films. Although the barrier and active functions can be enhanced, excessive disruption of the polymer network or interfacial structure may compromise the mechanical strength.

Furthermore, the method used to incorporate phenolic compounds also influences the active films. For example, when ferulic acid was sprayed onto PLA:PHBV/starch–gellan bilayers, the loading reached approximately 0.52 mg/cm^2^, and complete release occurred after approximately 6 h in an aqueous medium. The released ferulic acid concentration reached approximately 1250 mg/L, exceeding the reported MIC values for *Listeria innocua* and *Escherichia coli*. This enabled films to achieve approximately 2 log CFU reduction for *L. innocua* and 1 log CFU reduction for *E. coli*, while maintaining active and sealing properties for at least 2 months [95]. Similarly, PLA/starch/PLA trilayer films containing ferulic or cinnamic acid showed antibacterial activity against *E. coli* and *L. innocua*, with electrospun coatings being more effective than sprayed films because the active compounds were more efficiently exposed and released from the film surface [94]. These findings suggest that surface localization is particularly useful when rapid antimicrobial activity is required, whereas bulk incorporation may be more suitable for a slower or sustained release.

In extrusion-processed starch/PBAT systems, phenolic compounds are effective in developing active packaging films while maintaining acceptable processability. For instance, starch/PBAT blown films using tea polyphenols (TP) showed TP retention above 95% initially and above 80% after 12 months, indicating that a considerable fraction of the active compound survived melt processing and storage [96]. In the same system, WVP and OP decreased by approximately 15–20% and 25–30%, respectively, while TS and EAB slightly decreased. Additionally, the short-term TP release followed Fick’s second law, suggesting a concentration-driven diffusion mechanism rather than matrix swelling or degradation of the film. Such release behavior is important for active packaging design because Fickian release generally indicates a more predictable and controllable delivery of phenolic compounds into food simulants, whereas non-Fickian release implies a stronger dependence on matrix swelling, polymer relaxation, TPS hydration, or partial film disintegration [94,96]. Therefore, the Fickian behavior observed in this system suggests that tea polyphenol migration can be more reliably regulated by film thickness, phenolic loading, matrix morphology, and food-simulant polarity. Moreover, soil burial weight loss after 180 days reached about 60% for S/P-5TP, compared with about 50% for S/P-0TP and about 10% for pure PBAT [96]. This indicates that phenolic incorporation can contribute to active functionality without necessarily preventing biodegradation, although the degradation behavior still depends strongly on TPS content and film morphology.

The significance of TPS was further demonstrated in PBAT/TPS films incorporating tea polyphenols [97]. In these films, the water contact angle decreased from 104.25° to 91.35°, although the WVP increased by nearly 3.2 times. This increase in WVP was associated with the higher hydrophilicity and swelling of the TPS-rich phase. At the same time, TS and EAB decreased by approximately 70% and 31%, respectively. However, the increased swelling and diffusion promoted active functionality, with antioxidant activity increasing by 17–73% and inhibition zones increasing by 3.3-fold against *E. coli* and 2.4-fold against *S. aureus*. Soil-burial degradation also increased from 20% to 100% after 240 days, with the T30P70-TP film almost completely degraded after 180 days [97]. This highlights the main design challenges of polyester–starch active films. Higher TPS content can enhance the release, antimicrobial activity, antioxidant effectiveness, and biodegradation, but it may also weaken moisture resistance and mechanical strength.

However, several strategies have been used to reduce these trade-offs. For example, quercetin-containing PBAT/TPS films combined with organically modified montmorillonite showed improved antioxidant activity, UV shielding, and barrier properties. In this system, the oxygen and water barrier properties improved by up to approximately 94% and 54%, respectively, while the UV transmission decreased by approximately 50% [98]. Similarly, in starch/PBAT films containing blueberry extract, spraying the extract solution onto starch/PBAT pellets before blown extrusion helped preserve the heat-sensitive compounds. This approach retained 79.65% of polyphenols and 42.61% of anthocyanins, while TS and EAB reached 7.85 MPa and 606.53%, respectively [99]. The same films reduced O_2_ and CO_2_ permeability by 52.95% and 41.12%, respectively, and exhibited antioxidant and antibacterial activities of 68.69% and 72.40%, respectively. These results show that controlled incorporation strategies, such as pellet spraying, nanofiller-assisted dispersion, and encapsulation, can help maintain active functionality while limiting losses during melt processing.

Agro-industrial residues rich in phenolic compounds can serve as multifunctional additives in polymer matrices. In the PLA/cornstarch composite sheets, pomegranate peel powder (PGP) acted as both an antimicrobial and reinforcing filler. PGP-containing PLA showed inhibition zones of 10.42–25.17 mm against *S. aureus*, while combining starch with PGP further increased the inhibition zones to 18.62–33.20 mm [100]. This suggests that starch creates diffusion pathways for the release of PGP-derived phenolic compounds. In addition, at 10 wt.% PGP, the tensile strength increased from 31.3 MPa for neat PLA to 39.7 MPa. This example shows that phenolic residues may improve both the structural and antimicrobial properties when their particle rigidity, dispersion, and release behavior are well controlled.

Storage stability and end-of-life behavior are also important for packaging applications. PLA/starch laminates containing rice straw active extract released approximately 75–85% of phenolics after 1 week, and antioxidant activity was better retained in bilayer films [101]. These laminates maintained improved barrier stability during 10 weeks of storage, while all films were fully composted within 90 days. However, the active extract slowed the biodegradation rate, although it did not prevent complete biodegradation [101]. This is important because antimicrobial phenolic compounds may also influence microbial degradation during composting. Therefore, compostability claims should be linked to specific testing conditions rather than generalized to all disposal environments.

Beyond their active functionality, phenolic compounds have also been used to develop intelligent biodegradable polyester–starch films and extend the shelf life of packaged products. In PBAT/TPS films containing grape seed extract, the visible-light transmittance at 660 nm decreased from 11.65% to 5.82%, indicating improved visible-light shielding [102]. The antioxidant activity increased in a dose-dependent manner, and PBAT/TPS/GSE-5 extended the predicted shelf life of peanut butter to more than 300 days, approximately twice that of LDPE packaging. In another intelligent packaging system, blueberry anthocyanin–polyethylene oxide microcapsules were incorporated into PBAT/TPS films. Microencapsulation improved the thermal stability of anthocyanins, with *T*_max_ shifting from 310 to 400 °C. The film also showed 20.3% OP reduction at 2 phr BA-PEO, 62.83% TS improvement at 4 phr, and 85.6% DPPH scavenging activity, while shrimp freshness was visually indicated by the total color difference (ΔE*ab > 5) [103]. These studies demonstrate that polyester–starch films functionalized with phenolic compounds are progressing from simple active films to multifunctional systems capable of combining preservation, barrier improvement, optical response, and freshness monitoring. Nevertheless, translating these systems into food packaging requires careful optimization of phenolic loading, migration control, thermal stability during processing, food-contact safety, and validated composting behavior under typical end-of-life conditions.

## 5. Challenges and Future Perspectives

Despite the growing potential of biodegradable polyester–starch films functionalized with phenolic compounds, several technical challenges continue to limit their broader application in sustainable packaging. One of the main difficulties lies in the inherent incompatibility between hydrophilic starch or TPS and relatively hydrophobic biodegradable polyester phases. This often leads to weak interfacial adhesion, phase separation, unstable morphologies, and inconsistent mechanical and barrier properties of the films. These challenges can become even more complex after the addition of phenolic compounds, which may act as plasticizers, compatibilizers, crosslinking agents, or destabilizing components, depending on their structure, concentration, and method of incorporation. In addition, many phenolics are sensitive to heat, oxidation, and uncontrolled migration, which complicates their use in melt-processed systems and may reduce their functional efficiency during storage. During melt processing, phenolic compounds may lose their functionality through the oxidation of phenolic hydroxyl groups, volatilization of low-molecular-weight compounds, thermal decomposition, polymerization, glycosidic cleavage, or structural rearrangement. This concern is particularly important because PLA- and PHBV-based systems often require relatively high processing temperatures, whereas PBAT- and PBS-based films can be processed under comparatively milder conditions. For example, tea polyphenols, which show major degradation around ~250 °C, were successfully incorporated into starch/PBAT films by extrusion blowing, while TP retention remained >95% initially and >80% after 12 months [96]. In contrast, heat-sensitive anthocyanins showed degradation stages at 80–150 °C and 250–350 °C, but PEO microencapsulation shifted *T*_max_ from 310 to 400 °C, showing the stabilizing effect of encapsulation [103]. Ferulic and cinnamic acids in PLA/starch multilayers were applied by surface spraying or electrospinning to avoid prolonged thermal exposure and to place the active compounds near the food-contact surface [94]. Similarly, blueberry extract spraying before blown extrusion helped retain 79.65% of polyphenols and 42.61% of anthocyanins [99]. Therefore, matrix selection and incorporation strategies, including surface coating, pellet spraying, encapsulation, masterbatch dilution, reduced residence time, controlled thermal exposure, and low-oxygen or inert processing conditions, should be matched with the thermal stability of each phenolic compound.

Other important concerns include moisture sensitivity, release control, migration safety, variation in the composition of natural extracts, and the lack of standardized test methods for comparing antioxidant, antimicrobial, and barrier properties across different studies. For active food packaging, controlled release and migration safety should be considered together. The release of phenolic compounds is desirable when it contributes to antioxidant or antimicrobial activity at the food-contact interface, but excessive migration may raise safety, sensory, and regulatory concerns. Therefore, packaging films containing phenolic compounds should be evaluated through overall migration and, where relevant, specific migration testing using suitable food simulants selected according to the intended food type [107,108]. In the EU framework, Regulation (EU) No. 10/2011 establishes overall and specific migration requirements for plastic food-contact materials, while Regulation (EC) No. 450/2009 is particularly relevant for active systems because these materials may intentionally release substances into food or the package environment. In addition, FDA food-contact evaluation is generally use-specific and considers the intended application, expected migration or dietary exposure, and available toxicological information, while migration testing conditions should reflect the intended food type, contact time, temperature, and storage conditions [20,109]. For many naturally derived phenolics and plant extracts, fixed compound-specific migration limit values may not always be clearly available; therefore, safety evaluation should combine migration quantification, compound identification, exposure estimation, and toxicological justification [110,111]. Migration and release studies can be performed using analytical techniques such as HPLC-DAD, LC-MS/MS, GC-MS, and high-resolution mass spectrometry, depending on the target phenolic compounds, volatile migrants, and possible degradation products [112,113,114,115]. Moreover, improved biodegradability does not always imply real environmental benefits unless the end-of-life behavior is evaluated under realistic composting or disposal conditions.

In this context, end-of-life behavior should be interpreted cautiously because industrial composting and home composting occur under very different conditions. Industrial composting provides controlled thermophilic temperature, moisture, aeration, and microbial activity, whereas home composting is slower and less predictable. Consequently, findings from laboratory composting, industrial composting, or soil burial tests should not be directly generalized to uncontrolled disposal environments. Compostability standards such as ASTM D6400 and EN 13432 consider biodegradation, disintegration, and compost safety, whereas ISO 20200 and EN ISO 14855-1 are commonly used to evaluate disintegration and aerobic biodegradation under controlled composting conditions [30,101]. For example, Freitas et al. [101] reported that PLA–starch laminates were fully composted within 90 days using adapted ISO 20200 and EN ISO 14855-1 methods, although the PLA monolayer showed an approximately 35-day lag phase, and the active extract slowed the biodegradation rate without preventing complete degradation. In polyester–starch systems, TPS generally accelerates degradation by increasing water uptake, hydrolysis, and microbial accessibility, whereas higher polyester crystallinity and continuous hydrophobic domains can slow degradation [96]. Phenolic additives may also have dual effects by promoting fragmentation through increased hydrophilicity while locally delaying microbial mineralization due to their antimicrobial activity. Therefore, visual disintegration or mass loss alone should not be considered sufficient evidence of complete biodegradation, as incomplete mineralization may leave microplastic-like residues.

The environmental benefits of polyester–starch films functionalized with phenolic compounds should be evaluated beyond biodegradability and renewable feedstock origin. From life-cycle assessment (LCA) and techno-economic analysis (TEA) perspectives, their practical value depends on raw material sourcing, phenolic extraction or purification, drying, compounding energy, film-processing yield, cost of compatibilizers or encapsulation materials, shelf-life extension, food-contact safety, and the availability of suitable waste-management infrastructure. Their cost competitiveness with conventional PE, PP, or PET packaging also remains an important consideration. Recyclability may be limited by multilayer structures, starch-rich domains, phenolic additives, and food contamination, whereas compostable films may contaminate recycling streams if incorrectly sorted, and conventional plastics may contaminate composting streams. Therefore, the circular economy potential of these films should be supported by LCA, TEA, migration safety, toxicological assessment, compostability validation, sorting compatibility, and end-of-life evidence, rather than biodegradability alone.

Future research should focus on moving these materials from formulation-based studies to more application-focused and mechanistically informed designs. More attention is needed on compatibilization strategies, controlled release systems, and multilayer or encapsulation approaches that can preserve phenolic stability while maintaining the integrity and active functionality of the film. In parallel, more realistic packaging studies should be conducted under actual food storage conditions, including migration assessment, shelf-life validation and comparison with commercial packaging materials. The use of waste-derived phenolic extracts and starch from underutilized biomass also offers good opportunities to improve circularity and reduce costs; however, this will require better control over the extract composition and reproducibility.

From an industrial processing perspective, polyester–starch films containing phenolic compounds face several scale-up barriers. The hydrophilic TPS phase and hydrophobic polyester phase often exhibit melt-viscosity mismatch, weak interfacial adhesion, and phase separation, which can reduce bubble stability during blown-film extrusion and cause non-uniform film thickness. Residual moisture in starch or TPS may also promote hydrolysis during extrusion, whereas TPS retrogradation and moisture uptake during storage can change stiffness, extensibility, sealing behavior, and barrier properties. Therefore, industrial translation requires careful control of the drying, plasticizer content, screw speed, residence time, melt temperature, rheological matching, sealing window, and throughput. Direct extrusion is attractive for large-scale production, but multilayer design, compatibilization, encapsulation, or masterbatch-assisted incorporation may be required to maintain active functionality and film stability during continuous processing. Collectively, the future of biodegradable polyester–starch films functionalized with phenolic compounds depends on developing systems that are not only biodegradable and bioactive but also structurally stable, compatible with processing, safe for food contact, and sufficiently strong for sustainable packaging applications.

## 6. Conclusions

In the current food packaging scenario, there is a growing demand for packaging materials that can maintain food quality, extend shelf life, reduce environmental impact, and support safer preservation. Consequently, sustainable active packaging has become an important research focus in the packaging sector. In this regard, biodegradable polyester–starch films functionalized with phenolic compounds represent a promising class of sustainable active packaging materials. They combine the melt processability and structural integrity of biodegradable polyesters with the renewability and biodegradability of starch, as well as the multifunctional bioactivity of phenolic compounds. The present review shows that biodegradable polyesters, such as PLA, PBAT, PBS, PHAs, and related systems, provide a versatile material platform; however, their individual limitations in terms of cost, flexibility, barrier properties, and processing behavior often necessitate blending strategies. In this context, starch, particularly in the form of thermoplastic starch, has emerged as an attractive blending component because it can reduce formulation cost, increase renewable content, and enhance biodegradation behavior, although compatibility with polyester matrices and moisture sensitivity remain important challenges. The review further examines the beneficial role of phenolic compounds in biodegradable polyester–starch systems for imparting active packaging functions, particularly antioxidant and antimicrobial activities, while also affecting intermolecular interactions, morphology, and film properties. Recent studies have shown that the mechanical, barrier, optical, and active properties of these films depend strongly on the incorporation method, phenolic compound type, and blend composition. In summary, packaging films based on biodegradable polyesters such as PBAT and PBS are more suitable for direct melt processing of thermally sensitive phenolic compounds, whereas PLA-, PHBV-, and multilayer systems may require surface loading or encapsulation to better preserve active functionality. Additionally, reactive compatibilization and surface-localized phenolic delivery can improve film stability and release control, but their use should be balanced with processing complexity, migration safety, and storage stability. Collectively, the most promising systems are those in which material composition, compatibilization, and phenolic delivery are designed in an integrated manner to achieve a balanced combination of structural stability, active functionality, and shelf-life extension. Therefore, the functionalization of biodegradable polyester–starch films with phenolic compounds represents a promising pathway toward next-generation sustainable active food packaging, while also offering potential support for circular economy strategies and Sustainable Development Goals related to responsible consumption and production, climate action, and sustainable food systems.

## Figures and Tables

**Table 3:** Recent studies on biodegradable polyester–starch films functionalized with phenolic compounds for sustainable active packaging applications.

Polyester–Starch Film	Phenolic Compound	Incorporation Method	Key Characteristics	Active Functionality	Packaging Application	Reference
PLA:PHBV/cassava starch–gellan bilayer film	Ferulic acid, *p*-coumaric acid, protocatechuic acid	Phenolic acids incorporated into the polyester layer during melt blending; bilayers obtained by thermocompression	WVP reduced by ~53–65%; OP reduced by ~18–33%; TS decreased by ~25–33%; TBARS reduced by ~21–33% after 15 days; microbial reduction reached up to ~1.1 log CFU/g for total coliforms and ~1.2 log CFU/g for lactic acid bacteria.	Antioxidant and antimicrobial	Pork meat preservation	[93]
PLA:PHBV/cassava starch–gellan bilayer film	Ferulic acid (FA)	Surface spraying onto polyester sheet after bilayer formation	Ferulic acid loading was ~0.52 mg/cm^2^; complete release occurred after ~6 h in aqueous medium; DPPH EC_50_ was 0.172 ± 0.003 mg FA/mg DPPH; released concentration could reach ~1250 mg/L, exceeding reported MIC values for *L. innocua* and *E. coli*; growth inhibition was ~2 log CFU for *L. innocua* and ~1 log CFU for *E. coli*; active and sealing properties were stable for at least 2 months.	Antioxidant and antimicrobial	Active food packaging	[95]
PLA/starch/PLA trilayer film	Ferulic acid and cinnamic acid	Superficial incorporation by spraying 5% ethanolic solutions or electrospun PLA active coating	PLA/starch/PLA trilayers retained good mechanical and barrier properties; both spraying and electrospinning inhibited *E. coli* and *L. innocua*; cinnamic acid showed stronger antibacterial activity than ferulic acid; electrospun coatings were more effective than sprayed films due to better active-compound release.	Antimicrobial	Active food packaging	[94]
Starch/PBAT blown film	Tea polyphenol (TP)	One-pot pelleting followed by extrusion blowing	TP retention remained >95% initially and >80% after 12 months; TPC decreased by only ~10% after 12 months; DPPH scavenging capacity decreased by up to ~22% after 12 months. WVP and OP decreased by ~15–20% and ~25–30%, respectively, while TS and EAB slightly decreased. Short-term TP release fitted Fick’s second law, and soil-burial weight loss after 180 days reached ~60% for S/P-5TP compared with ~50% for S/P-0TP and ~10% for pure PBAT.	Antioxidant and antimicrobial	Active food packaging/food simulants	[96]
PBAT/TPS film	Tea polyphenol	Compounding and extrusion blowing	WCA decreased from 104.25° to 91.35°, while WVP increased ~3.2-fold. TS and EAB decreased by ~70% and ~31%, respectively. Swelling and TP diffusion increased markedly, enhancing antioxidant activity by 17–73% and inhibition zones by 3.3-fold against *E. coli* and 2.4-fold against *S. aureus*. Soil-burial degradation increased from 20% to 100% after 240 days, with T30P70-TP almost completely degraded after 180 days.	Antioxidant and antimicrobial	Controlled-release active packaging/food simulants	[97]
PBAT/TPS film	Quercetin	Extrusion blowing with organically modified montmorillonite-assisted formulation	Quercetin improved antioxidant and UV-blocking properties; OMMT improved mechanical, UV-barrier, gas-barrier, and water-barrier properties; oxygen and water barrier properties improved by up to ~94% and ~54%, respectively; polymer amount required for 50% DPPH inhibition decreased to 0.03 g; UV transmission decreased by ~50%.	Antioxidant, antimicrobial, and UV shielding	Banana and blueberry preservation	[98]
Starch/PBAT film	Blueberry extract	Blueberry extract solution sprayed onto starch/PBAT pellets before blown extrusion	BE spraying before blown extrusion retained 79.65% polyphenols and 42.61% anthocyanins; TS and EAB reached 7.85 MPa and 606.53%, respectively. O_2_ and CO_2_ permeability decreased by 52.95% and 41.12%, respectively; antioxidant and antibacterial activities reached 68.69% and 72.40%. The film showed visible alkaline color response (ΔE* ≥ 15) and pigment migration ≥ 70% in 50% ethanol.	Intelligent freshness indication, antioxidant, and antibacterial	Shrimp freshness monitoring	[99]
PLA/corn starch composite sheets	Pomegranate peel powder (PGP)	Composite extrusion sheet fabrication	PGP acted as both antimicrobial and reinforcing filler; pure PLA and PLA/starch showed no inhibition zone, while PGP-containing PLA showed inhibition zones of 10.42–25.17 mm against *S. aureus*. Combining starch with PGP further increased inhibition zones to 18.62–33.20 mm, indicating starch-assisted release of PGP bioactives. At 10 wt% PGP, tensile strength increased from 31.3 MPa for PLA to 39.7 MPa.	Antimicrobial	Food packaging sheets	[100]
PLA/starch laminate	Rice straw active extract	Active extract incorporated into PLA layer; cellulose fibers incorporated into starch layer; bilayers prepared by thermocompression	PLA–starch laminates showed improved barrier stability during 10-week storage; active extract or cellulose fibers reduced OP by ~41% and ~46%, respectively. Active films released ~75–85% phenolics after 1 week, with better antioxidant retention in bilayers. All films fully composted within 90 days; PLA showed ~35-day lag, while active extract slowed but did not prevent biodegradation.	Antioxidant	Compostable active food packaging films	[101]
PBAT/TPS film	Grape seed extract (GSE)	Blend extrusion and blow molding	GSE interacted mainly with TPS through hydrogen bonding; T_660_ decreased from 11.65% to 5.82%, indicating improved visible-light shielding. WVP and OP decreased with increasing GSE loading, while TS and EAB slightly decreased. Antioxidant activity increased dose-dependently, with GSE-5 showing nearly 5-fold higher activity than GSE-1. PBAT/TPS/GSE-5 extended peanut butter shelf life to >300 days, about twice that of LDPE packaging.	Antioxidant and antimicrobial	Peanut butter preservation	[102]
PBAT/TPS film	Blueberry anthocyanin–polyethylene oxide microcapsules (BA-PEO)	Microencapsulation followed by melt blending and film formation	BA-PEO showed 84.6% encapsulation efficiency; anthocyanin thermal stability improved with *T*_max_ shifting from 310 °C to 400 °C after encapsulation. OP reduced by 20.3% at 2 phr BA-PEO; TS increased by 62.83% at 4 phr. WVTR increased from 52.93 to 115.20 g·m^−2^·24 h^−1^, while DPPH scavenging reached 85.6% and shrimp freshness was visually indicated by a visible color difference (ΔE*ab > 5).	Intelligent freshness indication and antioxidant	Shrimp freshness monitoring	[103]

## Data Availability

No new data were created or analyzed in this study.
